# Supplementary material for: A Comparison of Three Measures to Identify Postnatal Anxiety: Analysis of the 2020 National Maternity Survey in England
Source: Int J Environ Res Public Health. 2022 May 28;19(11):6578. doi: 10.3390/ijerph19116578 (PMC9180011; doi:10.3390/ijerph19116578)
Supplement: Supplementary file 1 [file ijerph-19-06578-s001.zip › ijerph-1740781-supplementary.pdf]

Table S1: Response categories on the EPDS-3A and GAD-2

	**0**	**1**	**2**	**3**
**EPDS**				
3. I blame myself unnecessarily	No, never	Not very often	Yes, some of the time	Yes, most of the time
4. Feel anxious or worried for no good reason	No, not at all	Hardly ever	Yes, sometimes	Yes, very often
5. Feel scared or panicky for no good reason	No, not at all	No, not much	Yes, sometimes	Yes, quite a lot
**GAD-2**				
1. Nervous, anxious, on edge	Not at all	Several days	More than half the days	Nearly every day
2. Cant' stop or control worrying	Not at all	Several days	More than half the days	Nearly every day
